# 

**Published:** 1891-07

**Authors:** 


### TABLE OF CONTENTS.

ORIGINAL COMMUNICATIONS. PAGE

Anæsthetic Operation for the Treatment of Diseases of the Antral Chamber. By J. N. FARRAR, M.D., D.D.S., New York City . . . . . . . . . . . 421
Proper Care of the Eyesight. By LEWIS S. DIXON, M.D. . . . . . . . . 423
A Cursory Glance at Tetanus. By G. LENOX CURTIS, M.D., D.D.S. . . . 438
A Method of correcting Irregularities. By J. LEHMAN EISENBREY, D.D.S. 444
The Extraction of the First Permanent Molar. By DR. EUGENE J. WETZEL, Mulhouse, Germany . . . . . . . . . . . . . . . . . . . . 446
The Relation between Dental Caries and Diet. By L. ASHLEY FAUGHT, D.D.S., Philadelphia . . . . . . . . . . . . . . . . . . . . . . . . . 448
Secret Preparations. By DR. S. B. PALMER, Syracuse, N. Y. . . . . . . . 451
Local Anæsthesia and the Dentists. By DWIGHT M. CLAPP, D.M.D., Boston . . . . . . . . . . . . . . . . . . . . . . . . . . . . . . . 452
An Improvement in Flask Presses used within the Vulcanizer. By WILLIAM H. TRUEMAN, Philadelphia . . . . . . . . . . . . . . . . . 454

REPORTS OF SOCIETY MEETINGS.

New York Odontological Society . . . . . . . . . . . . . . . . . . . . 456
American Academy of Dental Science . . . . . . . . . . . . . . . . . . 466
New Jersey State Dental Society.—Twentieth Annual Session . . . . . . 470
Pennsylvania State Dental Society . . . . . . . . . . . . . . . . . . . 475
Harvard Odontological Society . . . . . . . . . . . . . . . . . . . . . 479

EDITORIAL.

Arsenious Acid in Dentistry . . . . . . . . . . . . . . . . . . . . . . 485
James W. White, A.M., M.D., D.D.S. . . . . . . . . . . . . . . . . . . 488
Saratoga Springs, New York . . . . . . . . . . . . . . . . . . . . . . 489
Southern Dental Association . . . . . . . . . . . . . . . . . . . . . . 489
Editor of the Dental Cosmos . . . . . . . . . . . . . . . . . . . . . . 489
Bibliography . . . . . . . . . . . . . . . . . . . . . . . . . . . . . 489

OBITUARY.

James W. White, A.M., M.D., D.D.S. . . . . . . . . . . . . . . . . . . 490
Chicago Dental Society—Resolutions on the Death of Drs. William H. Atkinson and James W. White . . . . . . . . . . . . . . . . . . . . 492
Resolution of the Central Dental Association, Northern New Jersey, on Dr. Atkinson . . . . . . . . . . . . . . . . . . . . . . . . . . . . 493
St. Louis Dental Society—Resolutions on the Death of Dr. Atkinson . . . 494
Dr. Ralph A. Parsons . . . . . . . . . . . . . . . . . . . . . . . . . 494

CURRENT NEWS.

The American Dental Society of Europe . . . . . . . . . . . . . . . . 495
New Jersey Examinations . . . . . . . . . . . . . . . . . . . . . . . 495
American Dental Association . . . . . . . . . . . . . . . . . . . . . 496
National Association of Dental Faculties . . . . . . . . . . . . . . . 497
Southern Dental Association . . . . . . . . . . . . . . . . . . . . . 497
Connecticut State Dental Association . . . . . . . . . . . . . . . . . 497
Alumni Association of the Philadelphia Dental College . . . . . . . . . 498
Alumni Association of the New York College of Dentistry . . . . . . . 498
Illinois State Dental Society . . . . . . . . . . . . . . . . . . . . . 498
List of Houses at Saratoga Springs, N. Y. . . . . . . . . . . . . . . . 499
American Dental Association . . . . . . . . . . . . . . . . . . . . . 499
College Commencement, University of Pennsylvania, Department of Dentistry . . . . . . . . . . . . . . . . . . . . . . . . . . . . . . 500
Union Meeting of the New Jersey and Pennsylvania State Dental Societies 500
Railroad Fares to the American Dental Association . . . . . . . . . . . 500

